# Factors of children's allocation behavior: peer relationship and resource quantity as the main determinants

**DOI:** 10.3389/fpsyg.2024.1368224

**Published:** 2024-06-04

**Authors:** RunYan Chen, Hao Zhu

**Affiliations:** School of Education Science, Hubei Normal University, Huangshi, Hubei, China

**Keywords:** resource allocation, peer relationship, resource quantity, equality, moral cognition

## Abstract

This study investigated the resource allocation of Chinese sixth-graders and the role of peer relationship in different resource conditions (*N* = 132, M_age_ = 11. 35 years, SD = 0.60). We designed the resource quantity as a between-group variable, with one group participating in a resource-limited experiment and another group in a resource-abundant experiment. Both groups of children allocated token resources to three types of peers relationships: good friends, disliked individuals, and strangers. Based on our experimental hypotheses, we presupposed three experimental outcomes: selfish allocation, equal allocation, and altruistic allocation. To analyze the data, we employed multivariate unordered regression analysis and performed two rounds of regression analyses using both selfish and altruistic allocations as reference categories to enhance the statistical power of regression model. Our results reveal that the resource quantity had a significant hindering effect on children's allocation behaviors, as the amount of available resources for allocation increased, so did their willingness to allocate selfishly. It was also found that an increase in resources led to a decrease in the proportion of children allocating equally. Nonetheless, the results still revealed generalized peer relationship preferences: children tended to allocate more resources to friends than to individuals they disliked. But when faced with disliked individuals, they were relatively more likely to allocate equally. Finally, we observed the proportion of equal allocation and discussed the similar impact of inequality aversion, different allocation contexts, and children's theory of mind on equitable allocation among sixth-graders.

## Introduction

Resource allocation tasks can examine psychological mechanisms such as children's norms of fairness, pro-sociality, moral development, and, more importantly, the pro-social competence represented by sharing will impact children's subjective wellbeing. Therefore, the search for “fairness preferences” has never waned in enthusiasm. Previous research indicates that children are able to share resources fairly and expect others to do the same (Shaw and Olson, 2012). The reason for this is that distributive fairness is a fundamental principle of human moral cognition, as both adults and preschoolers exhibit sensitivity to this concept. Infants as young as 2 years of age develop a basic understanding of the principle of fairness and loyal behavior and expect to be allocated resources fairly (Sommerville, 2018). While 3-year-olds display aversion toward unequal distribution and believe that equal distribution is the correct thing for a person to do (LoBue et al., 2011), 6-year-old children predicted that they would be able to share equally. Yet this group of children still tends to reserve more resources for themselves than for sharing equally with others in actual sharing behaviors (Smith et al., 2013). It is not until the age of 8 that children truly understand and apply the principle of fairness, when fairness perception and fairness behavior can be unified (Liang et al., 2015). As they mature, 9-year-olds engage in more altruistic behavior than 4-year-olds, and 14-year-olds make more altruistic demands on strangers in the ultimatum game than 7-year-olds (Harbaugh et al., 2003). It can be seen that advancing age causes children to demonstrate a heightened awareness of fairness norms and to be more attentive to meeting the needs of those to whom they are allocated.

However, is this really the case? Smith et al. (2013) has previously demonstrated experimentally that children subscribe to fairness norms associated with sharing, but when given the opportunity to share resources, their behavior often contradicts these norms. For example, Güroglu et al. (2014) found that 9 and 12-year-olds showed similar sharing behaviors for all interaction partners (including friends, disliked individuals, antagonists, neutral classmates, and anonymous partners), younger children didn't not show strong group preference, but the sharing behavior of older adolescents would change due to different peer relationships. This conclusion is also supported by the fact that as children understand social rules as they age, they are more and more deeply influenced by social and cultural norms (Jiang et al., 2022). Children tend to consider both group factors and principles of fairness when allocating resources in order to maintain stability in their social environment (Rizzo et al., 2017), which leads to complexity in children's resource allocation decisions and motivations. Children aged 9–12 years exhibit some degree of in-group favoritism, such as displaying greater resource sharing with acquaintances and friends or demonstrating strong loyalty toward close friends. While they also display prejudice and discrimination against out-group members (Everett et al., 2015). Nevertheless, research conducted by Elenbaas and Killen (2016) revealed that children between the ages of 10–11 tend to rectify unequal distribution through allocating fewer resources to in-group members. As students mature, their conception of fairness becomes contingent upon internal moral principles. Twelve-year-olds demonstrate the ability to make fair judgments based on factors such as “effort,” “achievement,” and “ability” (Fang and Wang, 1994). Absolutely, even infants are able to take into account the valence of distributive actions and expect agents to treat others in accord with their deservingness (Surian and Franchin, 2017). Acar and Sivis (2023) found that older children exhibit a fair allocation of resources by considering both demand for recipients and their received benefits, such as allocating more resources to those in need while distributing fewer resources to the rich. Zhang (2020) also revealed that children's resource allocation decisions are influenced not only by principles of fairness or group factors but also by moral cognition, psychological theories, and contextual factors. Therefore, studying resource allocation behaviors among sixth-grade children can effectively infer their motivations for allocation and investigate the influencing factors affecting sharing behaviors.

## Peer relationship

We believe that there is no group factor more closely related to the actual academic life of sixth graders than the peer relationships they construct through their interpersonal interactions. Peer relationships influence children's social behavior, social adjustment, academic performance and choice of peer conflict resolution strategies (Rubin et al., 2013), which naturally also affect children's decisions of resource allocation. Children will pay attention to the social identity of the recipient when allocating resources. For example, Hu (2021) found that both positive peer relationships (good friends) and negative peer relationships (disliked individuals) can affect the equal resource allocation of 4–6 year old children. However, we do not divide the experimental children's internal and external groups and examine their effects on children's distributive behavior. This is because in actual teaching, we found that children would still be close to and distant from their peers who belong to the same interest group or the same grade, so is the same group or the same grade not considered as an individual's “in-group”? Of course it is, but those who are alienated from the individual in the group cannot be regarded as members of the in-group. Typically, individuals usually take the initiative to establish closer connections with select members of these “in-groups,” gradually becoming good friends and expanding the number of friends step by step. This process helps them build “new in-groups” characterized by a stronger sense of belonging, where mutual love and sympathy among members prevail. Furthermore, it is highly likely for children to become future friends with people who were initially disliked in the in-group or strangers in the out-group. In the case of strangers, for example, who are members of the out-group for the child but still share common characteristics with the child itself (such as gender, age, and place of residence), it is challenging to assert that the child will not exhibit favoritism for strangers in their allocation behavior. Therefore, in our study, we did not narrowly define the group identity of recipients. Instead, we utilized children's friendship levels with recipients as a variable termed “Peer Relationship” in this experiment. The aim was to represent children's social relationships and allocation preferences based on social connection in real life.

## Resource quantity

While most research has demonstrated that children are more likely to allocate resources to their friends, it is not clear whether this sharing behavior is motivated by norms of fairness, generosity or friendship. So if resources are limited, or if the needs of each person to be allocated cannot be met, will children's generosity still be higher than the friendship-constructed in-group preference? Therefore, whatever the psychological motivation, it is indispensable to discuss the influence of the characteristics of the allocation situation on children's sharing behavior. We argue that although the degree of relationship with the recipient is a prerequisite for children's sharing behavior with in-group members, but friendship or generosity is not the only predictor of their sharing behavior with out-group members. The important role of resource quantity in allocation behavior is also of interest. In studies where tokens (which can be exchanged for money and gifts) are used as an allocative resource, children ages 7 allocate 3.5% of their tokens to recipient, while 9- to 14-year-olds allocate about 13% of their tokens to recipient (Harbaugh et al., 2003). In addition, Kenward and Dahl (2011) involved children ages 3–4 in experiments with non-interest-involved situations, and they revealed that from age 4 onwards, children's sharing behaviors were influenced by a combination of the resource quantity and recipient traits. In previous research on distributive justice that further explores the amount of resources and recipients' characteristic, it has been observed that children tend to distribute resources equally among all recipients when the quantity of resources matches the number of recipients, without considering camaraderie or reciprocity principles. The first consideration of peer relationship to distribute is revealed when the child discovers that there are insufficient resources to distribute an equal amount to each potential recipient (Geraci and Surian, 2011). In summary, the majority of children observe the principle of equality when there are just enough resources for two recipients to share, however, when the distributing singular or indivisible resource, they exhibt in-group loyalty (Lee et al., 2018). The differential allocation outcomes resulting from varying resource quantities can be attributed to the distinct emotional responses evoked by difference amounts. Similar to an investor would prefer a business with ample resources over one with limited resources, sufficient resources will provide individual a sense of security and pleasure in order to further act comfortably. As Feigenson et al. (2002) also noted, people favor abundant resources (a jar with many balls) to scarce resources (a jar with only one ball). However, no scholars have discussed whether children's allocation behavior in the sixth year of primary school is affected by both the resources quantity and the nature of the distributors. For instance, when the allocated resources are limited, do children's equal allocations still show peer relationship preferences, or does children's consistent preference for best friends change depending on the number of resources? In this study, we argue that children's allocation behavior is not only influenced by different peer relationships, but also by the resource quantity, or by the combination of this two. We expect that children's allocation behavior may take on new patterns.

## The present study

In the present study, we tested how resource quantity and peer relationship would influence children's allocation behaviors. We also investigated how peer relationships and resource quantity affected children's allocation behavior through the observed allocation results, as well as examining the extent to which the two independent variables influenced the dependent variable. Just like suggestions of Geraci et al. (2023), the research direction of future development should put forward a methodology with different variables and more interaction between different variables. The experiment used a mixed experimental design, with one group of 66 children participating in the resource-limited group making resource allocations, and another group of 66 children participating in the resource-abundant group allocating resources. We predicted three possible outcomes for children's allocations: selfish allocation, altruistic allocation, and equal allocation. Detailed criteria for classifying these outcomes are described in the *Coding strategy* section. Notably, there are three possible allocation outcomes, we focus our discussion on equal allocation as well. Apart from discussing the effects of resource quantities and peer relationships on children's resource allocation, we will also discursively discuss other factors that influenced equal distribution in this experiment.

We entered the school early to gain insight into children's genuine perspectives, they simply categorize their relationships as good friends, acquaintances, classmates they dislike, and individuals they have yet to know. Combined with previous classifications of peer relationships, Cui (2011) divided peer acceptance and peer rejection based on group level, and Newcomb et al. (1993) divided five categories of peer relationships: popular children, rejected children, controversial children, neglected children, and children of general status. In our study, we pre-defined three potential peer relationships for children. Prior to conducting the experiment, we employed a peer nomination method to obtain children's 2 types of peer relationships by asking each child to write down names on cards for “close friends in grade” and “annoying people in grade.” Which allowed them to select both a friend and someone they dislike from in-group members: Good Friend, Disliked individual. And our researcher nominated an out-group member as stranger.

It is now more common to measure prosocial behavior using resource allocation or ultimatum and dictator games, instead of relying solely on traditional questionnaires or combining brain neuroscience to detect the degree of pro-social development of an individual. Specific research paradigms have used interest involvement perspectives to construct scenarios of interest confrontation. Some researchers even used the results of resource allocation as a cue to infer the strength of the social relationship between the distributor and the recipient (Shaw et al., 2012). It has been found that children over the age of 8 years can show the pursuit of fairness and the aversion to unfairness regardless of interest-involved or non-involved conditions (Liang et al., 2015)). But compared with non-interest-involved situations, children's egoism may be stronger in interest-involved conditions because fair distribution involves more personal cost, especially when they find out that they have received less benefits than others, and they will show a strong disadvantageous inequity aversion (Güroğlu et al., 2014). Therefore, we asked children to participate in a resource allocation task in the form of interest involvement to visibly present the differences in children's allocation decisions when facing different peer relationships in difference resource quantities.

A further important question is controlling the value of token exchange objects since children tend to assess an item's worth based on its objective attributes (Chernyak and Sobel, 2016). Choshen-Hillel et al. (2020) discovered that higher value led to more efficient allocations by children, which revealed the resource value would affected the children's experimental responses. The experimental results of Echelbarger and Gelman (2017) demonstrated that both adults and children typically choose items based on variety and necessity, without knowledge of the item's own value. It can be seen that both the resource quantity and value impact children's allocation behavior. Therefore, in order to prevent children's subjective judgement of resource value from interfering with the results of the experiment, we designed token with low value as exchanges. In the specific experiment, we gave children tokens to allocate among three peer relationships, and these children could exchange the tokens for stickers at the end of the experiment. Cartoon stickers, although cute, are not useful for anything other than decorative purposes.

Based on the literature reviewed above, we formulated the following hypotheses: (1) Resource quantity and peer relationships significantly would affect children's allocation behaviors. (2) When resources quantity was abundant, children would make more altruistic and equal allocations. When there were limited resources, children would make more egoistic allocations. (3) Regardless of the resource quantity, children in grade 6 will give more resources to good friends, less resource to disliked individuals, and make more egalitarian allocations toward strangers.

## Methods

### Participants

Excluding nineteen students who were unable to provide valid experimental data due to personal or external reasons, a total of one hundred and thirty-two participants (*N* = 132, *M*_*age*_ = 11.35 years, *SD* = 0.60; 61 females) finally participated in this experiment. None of the students had ever participated in a similar experiment before. Participants were recruited from a local primary school in an urban area of Hubei, China. All data was collected from October 2023 to November 2023.

### Materials

We set the number of resources to an odd number and put an opaque “extra bucket” (Renno and Shutts, 2015). This was done to provide children with the opportunity to make an equal allocation of the discarded resources. Individual socially based allocation behavior is better demonstrated in situations where an egalitarian solution cannot be given quickly, especially when there are two potential recipients but an odd number of resources (one or three) to allocate. Therefore, we design the between-group variable: one token is the resource-limited group and three tokens is the resource-abundant group. The experimental materials were: a certain number of plastic chips as tokens, an extra bucket in which resources could be wasted, six dolls with converging images, and a certain number of reward stickers.

### Procedure

The primary researcher initially entered each classroom and informed children participating in the upcoming experiment. Subsequently, children who had agreed to participate in the experiment would enter the designated experimental site in an orderly manner. Each participant was tested individually by a female experimenter in a closed, separate room within the school. The researcher asked children to complete peer nomination cards and promised to keep their responses confidential. After collecting peer nominations, each child would was instructed to allocate resources to one recipient, who could be their best friend, disliked individual, a stranger. However, in order to examine children's natural allocation beliefs arising from non-interaction, children were told to treat the first and second dolls as peers for the different relationships just written (good friend, disliked individual) respectively. Afterwards, the researchers introduced an opposite-sex students in the same grade from another elementary school to children, thereby establishing a stranger relationship, and the stranger was replaced by the third doll.

The resource-limited scenario was designed so that the resource can only satisfy the needs of one person, while the resource-abundant scenario was designed so that the resource could be divided equally between two individuals with a surplus resource. During the experiment, the researcher provided each participant with one (or three) token (s), and first pointed to “Doll 1,” who represented as their “best friend.” researcher explained to the children: “Now that you have 1 (or 3) tokens, please think of 'Doll 1' as your best friend. Please distribute these tokens. You can distribute them to yourself, to each other, or if you don't want to distribute them to anyone, you can choose to discard them in this bucket.” The second and third doll were then would replace with representations of disliked individual and stranger. After determining the allocation of resources to each doll by the children, the researchers recorded the distributive outcomes.

It was important to note that the main researcher would tell each pupil in the experiment in advance that they must allocated all tokens in allocation task at a time, and could not keep one for the next recipient or kept it in their own hands (For example, some child might know that the next recipient to be assigned is their good friend, so when assigning resources to someone they disliked, they might intentionally save one for their good friend, which was not allowed). At the same time, whenever a participant used an “extra bucket,” the researcher should inquire about their rationale for equitable allocation as well as the reasons for discarding resources, and recorded them.

### Coding strategy

To classify the type of distribution results in this experiment, this study mainly referred to the scoring method of Kang et al. (2023)'s experiment, which awarded one point for each item distributed by children. They also compared the number of distributions by the distributor and the recipient, so as to classify the three types of distributions: Equal distribution was defined as when children distributed an amount equal to half of the allocated material; altruistic distribution occurred when more than half of the allocated material was distributed; selfish distribution happened when less than half of the allocated material was distributed. However, since it was not possible to calculate tokens in halves in our study, we only compared the higher or lower number of tokens owned by both parties to classify allocation results. Eventually, in our study, we designed the allocation results to present three kinds of results: Selfish allocation (coded as 0) when the number of tokens received by subject A > the number of tokens received by object B. Equal allocation (coded as 1) when the number of tokens received by subject A = the number of tokens received by object B. Altruistic allocation (coded as 2) when the number of tokens received by subject A < the number of tokens received by object B. In addition, in order to study efficiency, Peer relationship variable, in coded form (1 = good friend, 2= stranger, 3 = disliked individual); Resource quantity variable, in coded form (1 = resource-limited, 2 = resource-abundant).

## Results

### Differential comparison between peer relationship and resource quantity on children's allocation behavior

Since both independent and dependent variables are not continuous data and belong to categorical variables, so the chi-square test was employed to examine the independence and difference among these categorical variables. The count data were presented as percentages, with partial correlation analyses used to determine the direction and magnitude of the correlation, followed by multivariate logistic regression with α = 0.05. The results of the chi-square test revealed that children's overall distributional behavior in terms of allocation is predominantly selfish (41.4%), followed by altruistic (37.9%), and the least equal allocation (20.7%).

**Figure 1:**
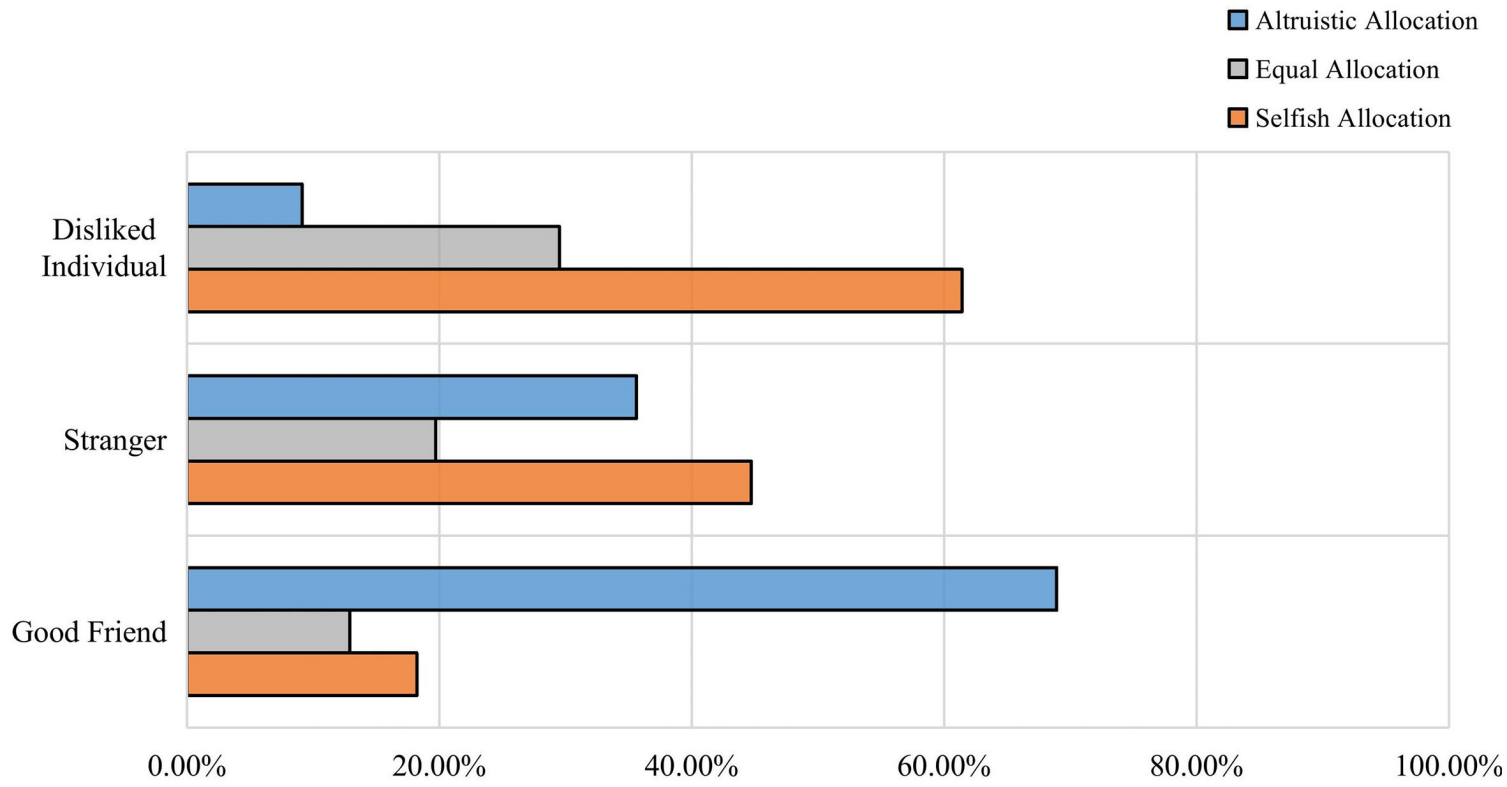
The proportion of children's allocation behavior to different peer relationships.

There was a significant effect of peer relationships (χ^2^= 101.86, df = 4, *p* < 0.001, ϕ = −0.47). And children made more altruistic allocations toward their good friends and more egoistic allocations to their disliked individuals, children made relatively greater proportions of selfish allocation when confronted with strangers. After controlling for resource quantity, partial correlations between peer relationship and children's allocations were significant strong negative correlation (ϕ = −0.50, *p* < 0.001). There was a strong increase in children's tendency to make selfish allocations as they became more detached from their peers (see Figure 1).

**Figure 2:**
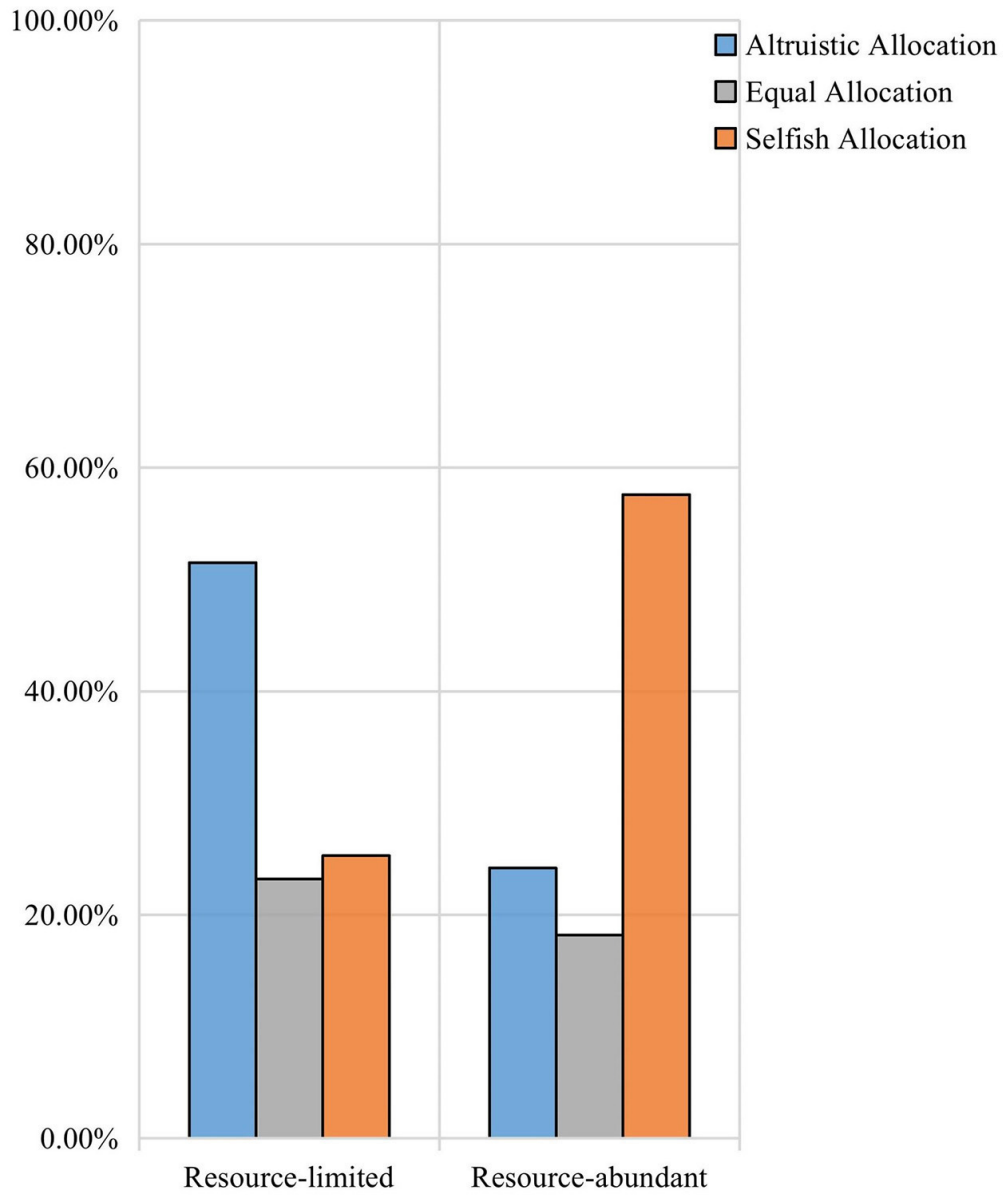
The proportion of children's allocation behavior in different resource quantities.

The chi-square test also showed a significant effect of resource quantity on children's allocation behaviors (χ^2^= 45.64, df = 2, *p* < 0.001, ϕ = −0.34). Children in the group with abundant resources were more willing to make altruistic allocations than children in the group with limited resources. At the same time, children tended to make equal allocations in the context of limited resources. After controlling for peer relationship, partial correlations between resource quantity and children's allocations were significant general negative correlation (ϕ = −0.38, *p* < 0.001). Indicating that the increase of selfish allocations were small to moderate in magnitude when the resources were limited (see Figure 2).

Additionally, we investigated whether there was an interaction between resource quantity and peer relationships on children's allocation behaviors, we used the 2^*^3 Crosstabs analysis. We found there was no significant combined effect of these two variables on children's allocation behaviors in our study (χ^2^= 1.39, df = 2, *p* > 0.05; χ^2^ = 1.39 < 5.99, 2-tailed chi-square at the 95% confidence intervals). Considering that both variables independently had a significant impact on allocation behaviors (*p* < 0.001), we concluded that there is no interaction between resource quantity and peer relationship.

### Differential comparison of dropping resources for equal allocation

**Figure 3:**
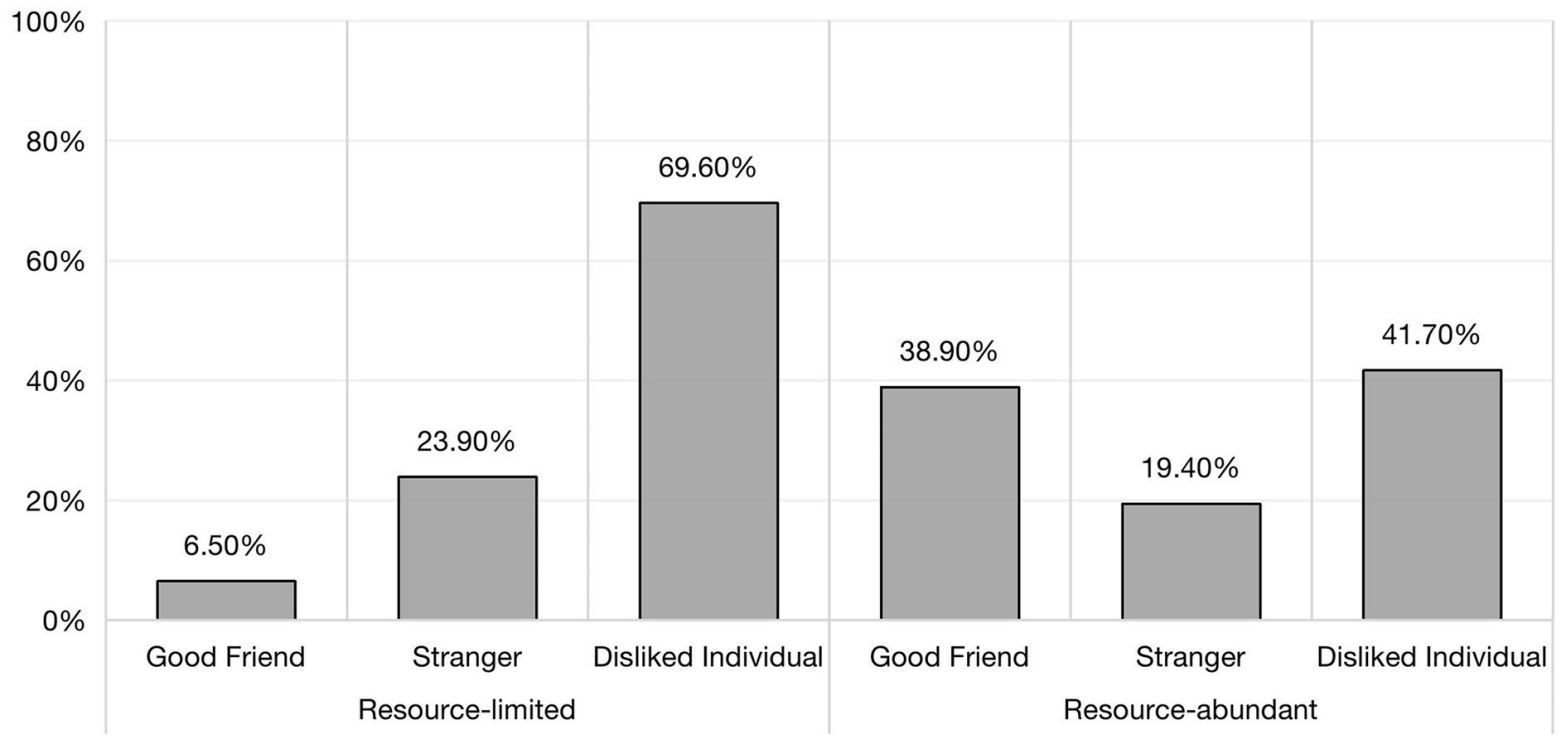
The proportion of equal allocation of children facing different peer relationships in different resource quantities.

Children participated in our trials featuring limited or abundant resources and three peer relationships, and had the option to use an extra bucket to achieve equal token distribution (some participants did). The aforementioned to, wasting resources to achieve equal allocation was only 20.7%, and further chi-square results indicate that the number of resources and peer relationships had a significant effect on children's equal allocation (χ^2^ = 13.13, df = 2, *p* = 0.001). Notably, children's tendency to make equal allocations toward disliked individuals was most pronounced regardless of the resource quantities (see Figure 3). When children faced with disliked individuals, those in the resource-limited group were more likely to discard resources in order to make an equal distribution (70%), compared to those in the resource-abundant group (42%). These findings suggested that children with limited resource demonstrate a greater willingness to sacrifice resources in order to achieve equality, especially when confronted with disliked individuals.

### The impact and degree of peer relationships and the amount of resources on children's allocation behavior

To examine the impact of resource quantity and peer relationship on children's allocation behavior, resource quantity was made a factor and peer relationship was included as co-variate in the logistic regressions (Tables 1, 2). Table 1 presents the logistic data affecting children's selfish and equal allocations, using altruistic allocation as the reference category for the dependent variable. “Peer relations-3” (disliked individual) is the reference category in the corresponding independent variable, thus assigned is 0.

**Table 1:** Multiple logistic regression results influencing children's sharing behavior (altruistic allocation as benchmark).

	**Selfish allocation**			**Equal allocation**		
	**B (SE)**	**Exp. (B)**	**95% CI**	**B (SE)**	**Exp. (B)**	**95% CI**
Resource quantity	2.168^***^	8.738	4.776-15.985	1.005^**^	2.732	1.446–5.164
	(0.288)			(0.325)		
Peer relationship-1	−3.815^***^	0.022	0.009-0.052	−3.121^***^	0.044	0.019–0.105
	(0.434)			(0.443)		
Peer relationship-2	−1.913^***^	0.148	0.068-0.319	−1.868^***^	0.154	0.068–0.349
	(0.393)			(0.416)		
Peer Peer relationship-3	0^a^	/	/	0^a^	/	/

^**^p < 0.01, ^***^p < 0.001. Dependent variable is children's allocation (selfish allocation, equal allocation, altruistic allocation), Peer relationship-1 = good friend, Peer relationship-2 = stranger, Peer relationship-3 = disliked individual. ^a^Peer relationship-3 is the reference category of the independent variable.

**Table 2:** Multiple logistic regression results influencing children's sharing behavior (selfish allocation as benchmark).

	**Equal allocation**			**Altruistic allocation**		
	**B (SE)**	**Exp. (B)**	**95% CI**	**B (SE)**	**Exp. (B)**	**95% CI**
Resource quantity	−1.162^***^	0.313	0.178–0.550	−2.168^***^	0.114	0.063–0.209
	(0.288)			(0.308)		
Peer relationship-1	0.693	2.001	0.932–4.293	3.815^***^	45.370	19.361–106.318
	(0.390)			(0.434)		
Peer relationship-2	0.045	1.046	0.562–1.984	1.913^***^	6.771	3.136–14.619
	(0.317)			(0.393)		
Peer relationship-3	0^a^	/	/	0^a^	/	/

^***^p < 0.001. ^a^Peer relationship-3 is the reference category of the independent variable..

When altruistic allocation was used as a reference, resource quantity had significant contributory effects on children's allocation behavior. Compared to altruistic allocation, an increase in resource quantity made children 8.7 times more likely to engage in selfish allocation rather than altruistic allocation, and children were 2.7 times more likely to make equal allocation than altruistic allocation. This implied that children tend to exhibit selfish or equal allocations when resources were abundant. And tend to make altruistic allocation when resources were limited. Additionally, peer relationships had a significant hindering effect on children's allocation behavior. Compared to altruistic allocation, children made a selfish allocation to a good friend was 0.02 times higher than that to disliked individual, and made selfish allocation to a stranger was 0.15 times higher than that to disliked individual. Moreover, Compared to altruistic allocations, the probability of making an equal allocation to a good friend was 0.04 times higher than to disliked individual, and the probability of making an equal allocation to a stranger was 0.15 times higher than to disliked individual. The implication was that children demonstrate greater inclination for selfish or equal allocations when faced with disliked individuals and strangers; however, when confronted with good friends, their tendency shifted toward altruistic allocations (see Table 1).

To enhance the predictive capability of the logistic regression model in estimating probabilities, we also incorporated selfish allocation as a benchmark (refer to Table 2). Similar to the results in Table 1, but we unexpectedly found that allocating to good friends, strangers and disliked individuals did not yield significant effects on children's equal allocation (*p* > 0.05). Which indicated that peer relationships did not exert a substantial influence on children's fairness judgments.

## Discussion

### The role of peer relationship

Maintaining strong interpersonal relationships is crucial to children's survival, so children attach great significance to constructing harmonious peer relationships. Children exhibit a stronger preference for good friends due to their need for social recognition and individual identity within the group. Scientists who study human relationships claim that the motivations, emotions and communication involved in different relationships affect an individual's social cognition and behavior (Reis et al., 2000). Thus, by observing resource allocation decisions among sixth grade students, it is possible to examine their understanding of fairness in different relationships. As previous research on children's social information processing (SIP) has posited, the affective value of relationships influences how children process and interpret information, which subsequently affects how they make their final behaviors in these relationships. The results of our study also demonstrated that peer relationships have a significant impact on children's allocation behaviors (*p* < 0.001). Consistent with the SIP researchers' results, distinct peer relationships exhibit significantly different affective profile, displaying positive affect toward friends, weak or neutral affect toward acquaintances, and strong negative affect toward disliked peers. These emotional dispositions correspond behavioral motivations and intentions (Peets et al., 2008). The results of partial correlation analysis and logistic regression support this view, we coded peer relationship-3 as the people children dislike, and peer relationship-1 as children's good friends, the results were presented as peer relationships had a significant hindering effect on children's allocation behavior. In other words, when faced with recipients who are more distantly related to them socially, children tend to make selfish allocations. The reality also is that children are more generous in allocating resources to their good friends (Kang et al., 2023), while exhibiting self-centered tendencies toward disliked individuals due to children will avoid benefiting disliked individuals. In our experiment, we also observed that sixth-grade students did not exhibit significant altruistic or equal tendencies when they allocated the resources to strangers. This can be attributed to the fact that children's allocation to strangers depends on whether it incurs personal costs (Moore, 2009). When faced with a stranger, children inquire about their characteristics and needs in order to comprehensively consider how much they should give. It's worth mentioning that I found unexpectedly that the proportion of children making equal allocation to good friends and disliked individuals did minimal difference (refer to Figure 3), meaning that children paradoxically showed a sense of equality in the face of disliked individuals as well. In general, children make equal allocations regardless of their social relationship with the recipient (Olson and Spelke, 2008). Our study not only supported this view, but also confirmed the developmental view of social reasoning, in which a subset of sixth-grade students viewed “fair distribution” as crucial for establishing and maintaining harmonious interpersonal relationships, including ensuring that disliked individuals were treated with equal respect and treatment.

In summary, peer relationships are an important influence on children's allocation behaviors, and as they aged, children increasingly prioritize social relationships when allocating resources (Smetana and Ball, 2018). Children tend to give more to good friends and less to disliked individuals. We have verified the special role of friendship in children's sharing behavior (Güroglu et al., 2014), and its role even outweighs the sense of fairness of some sixth students. However, some children will show more fairness to disliked individuals compared to good friends and strangers, suggesting that peer relationships may not be the sole predictor of children's allocation behaviors, and that a proportion of children have acquired the ability to suppress group favoritism to modify unequal outcomes.

### Resource quantity

We devised two resource quantity scenarios, the limited resource scenario, where resources were sufficient for only one person's needs, and the abundant resource scenario where there was a surplus beyond meeting the requirements of both parties. By comparing children's allocation decisions in the two context, we observed children's equity preferences across peer relationships with different resource quantities. Surprisingly, our partial correlation analyses revealed a significant negative association between the resource quantity and sixth-grade children's allocation behaviors. At the same time, logistic regression results suggested that an increase in resource quantities significantly contributed to children's egalitarian and selfish allocation compared to altruistic allocation. This implied that children are more likely to allocate altruistically when the number of resource is limited and more likely to make selfish allocations when the number of resource is abundant. Compared with the two resource quantities, children were more willing to make equal distribution when resource was limited. Although the result was contrary to our previous hypothesis, it still corroborated some of the previous findings that children preferred rare (vs. abundant) resources when allocating resources to favorite puppets (Chernyak and Sobel, 2016). Or children perceive limited supplies as preferable, when it comes to consuming more limited food or playing with scarce toys for extended periods (Huh and Friedman, 2019; Maimaran and Salant, 2019). Therefore, on the premise that the amount of resources can satisfy the needs of both parties, how the remaining resources are divided becomes less crucial (Sui et al., 2023). According to the law of diminishing marginal utility theory, after equal sharing of resources among peers is achieved, wasting any remaining surplus becomes undesirable for children, their inherent self-interest motivation which drives them toward self-serving behavior in situations characterized by abundant resources. In addition, this experiment found that when peer relationships and resource quantity influence children's allocation behavior, the amount of resources amplifies their peer's preferences. Consistent with Olson and Spelke (2008) study, children prioritize relatives, friends, returners and reciprocators over strangers when there resources are insufficient to meet everyone's needs. Notably, children exhibit strong in-group loyalty when allocating involves singular, indivisible resources (Renno and Shutts, 2015; Lee et al., 2018). Thereby displaying altruistic tendencies in contexts characterized by limited resources and more selfish allocation behaviors in situations of resource abundance. This finding partially supports the realistic group conflict theory (Jackson, 1993), where striving for limited resources will exacerbate competition and conflict between groups. Historically, tribes worked collaboratively as teams where chiefs assigned tribal members acquire and protect valuable resources in order to avoid receiving hostile damage from external invaders (Benozio and Diesendruck, 2015). Finally, we suspected that these results may be attributed to the resource quantity to be allocated in the experiment was still not large enough, and that 3 tokens was not a lot for children compared to 1 token with limited resources, “limited” or “abundant” were just hypothetical experimental scenarios. Most children couldn't afford to throw away the resources, so they were more inclined to choose the allocation behavior that sacrifices fairness but did not waste it, so the proportion of equal allocation was very low.

In conclusion, how to allocate resources is an important aspect of human life, especially now when material resources are limited. Outward sharing behavior serves as an important indicator of children's ethical conduct, while sharing decisions are associated with individual differences in psychological motivation, cognitive factors, and social relationships. Children who are able to make more altruistic allocations in resource-limited scenarios will demonstrate better prosocial competence, and these children will have higher subjective wellbeing (Aknin et al., 2012), overall mental health levels, and interpersonal trust, which will help to stabilize their social existence.

### Other reasons on equal allocation

In this study, we see some results of equal distribution, the chi-square test shows that the proportion of equal distribution is only 20.7% though. Indicating that the majority of participants neglected the slightly extra bucket in most trials and also ignored the principle of fairness. Interestingly, children exhibited the highest probability of making equal allocations in the face of disliked individuals, at 57.3%, and the reason for this situation intrigued us. Previous research has found that although children in Grade 6 still show some group favoritism, those who value distributive justice weigh up social relationships and moral norms to allocate resources fairly, for example, some children rectify inequalities by allocating additional resources to out-group members (Elenbaas and Killen, 2016), it is evident that it is not only the quantity of resources and peer relationships that influence children's allocations, but there may be other potential factors that act simultaneously to influence children's equal allocation outcomes. Therefore, we would like to continue to discuss the reasons for the above results in this experiment, and this study suggests that there are the following: inequality aversion, different allocation contexts, and the level of theory of mind which to a certain extent also affect children's equal allocations.

Firstly, People usually use fairness as their moral principle, leading individuals to strongly reject inequality. In some societies, when older children or adults are given more resources than their peers, they will choose to discard the extra resources to achieve equity for both parties (Corbit et al., 2017). This has been described as inequality aversion and takes two forms, One preference not to have access to more resources than one's peers, called advantageous inequality aversion, this is mostly attributed to a consideration for fairness and an expectation of maintaining positive reputation (Fehr and Schmidt, 1999). Children's reasons for equal allocation include statements such as:

I don't want to appear too selfish (male, 11).

I'm afraid she'll be too envious of me (female, 11).

I still want to play with him, even though I hate him (male, 12).

Everyone is equal, if he does not possess it, neither should I (male, 11).

I might not hate him so much in the future (male, 11).

The other is a preference not to receive fewer resources than peers, called disadvantageous inequality aversion. This is mostly attributed to psychological mechanisms of selfishness, envy, and resentment (McAuliffe et al., 2014). The children's reasons for equal allocating such as:

I do hate him (female,12).

I refuse to give him anything (female, 11).

I'd hate to get him out of the world (female,11).

Since I detest him so much, I shall withhold it from him (male, 11).

Discard it rather than giving it to someone I hates (male, 11).

It is worth noting that it is still an open question whether an equal distribution influenced by inequality aversion is fair or not, since people may distribute fairly to avoid inequality in order to avoid partiality (Shaw and Olson, 2012).

Secondly, the mode of situational involvement prominently affects children's distributive equality, with children showing more inequality aversion to unfair outcomes in the non-interest-involved condition than in the interest-involved condition. The reason for this is that in the interest-involved condition, it is expensive for people to sacrifice resources to maintain equality (Shaw and Olson, 2012). Early adolescents' decisions are more impacted by perceived benefit (Franchin et al., 2023), so when children act as stakeholders in the distribution of resources, primitive selfish motives allow them to readily accept more for themselves while not accepting more for strangers. Therefore, in interest-involved scenarios, children as allocators show in-group favoritism (Fehr et al., 2008). In contrast, in non-interest-involved scenarios, where children's self-interests are not involved, children are more likely to make equality distributional decisions and are less affected by in-group favoritism (Rochat et al., 2009). Even in the same interest-involved scenarios, differences in allocation paradigms can affect children's fairness. The results of Sui et al.'s (2023) study present that children aged 10 and 12 years old will make more altruistic assignments in first-party allocation situations due to children's decreased self-service motivation. However, in some experiment, which also involved children around the age of 12 years old entering first person case for an allocation task, resulted in a greater proportion of children making selfish allocations. This is due to the different allocation task paradigms between the two experiments. When children were asked to give a direct allocation result, children considered the social relationship with the recipient more than fairness (Li et al., 2019). And when children were offered multiple distributional paradigms and chose one of them as a distributional decision, children more often chose equality option.

Third, theory of mind is an ability to attribute mental states such as desires, intentions, beliefs and needs about oneself and others. It is an crucial social-cognitive basis for pro-social behavior in distributive gaming. So theory of mind as a social cognitive ability overlaps with the developmental trajectory of children's distributive equality. As children develop higher levels of cognitive experience, theory of mind is also more refined to make a fairness consideration that allows them to respect for others while maximizing their own interests (Chen and Wu, 2017). The results of the above experiments showed that there was no significant selfish or altruistic tendency in children's allocation of outcomes in the face of strangers. This is because the relationship between children and strangers is one of exchange, that children are not responsible for the interests of strangers, and that there is no norm of equal distribution (Yu et al., 2016). In real life, sixth-grade children often need to understand what their peers lack or deserve during social interactions. Therefore, many sixth-grade children ask researcher questions before distributing tokens to strangers such as “Does she like stickers?” “I can give it to her if she really needs it” “Will he be my friend if I give it to him?” Although children do not have responsibility for others' wellbeing and for making fair norms in the face of strangers because they are out-group members, children with a strong theory of mind can “put themselves in the shoes of others.” In contrast, the lowest proportion of equal allocations was made with friends, precisely because children's relationships with friends are communal and children have a sense of responsibility for the interests of their friends, and they do not need to apply theory of mind in order to make allocations based on pre-existing norms and experiences. It is also due to this sense of responsibility for their friends, and because the theory of mind allows the sixth graders to focus on their friends' immediate interests, that the in-group preference in the allocation process leads to a more altruistic allocation. In conclusion, the reality of the experiment makes us realize that children's psychological mechanisms for equal allocation may be rooted in maintaining interpersonal relationships, the expectation of higher rewards, subtle societal cultural influences, empathetic understanding of others, and the result of socialization through education. This all represents an important role theories of mind in children's distributive equality. Therefore, future studies should not limit themselves to examining independent variables but also explore other psychological factors within children using sophisticated empirical methods.

## Conclusion

This study reveals a strong correlation between sixth-grade students' allocation behavior and their peer relationships as well as the quantity of resources. The specific conclusions are as follows: (1) Sixth-grade students' allocation behavior was affected significantly by peer relationships, and they tended to give more resources to their good friends and fewer resources to their disliked individuals, and among the three kinds of peer relationships, children's tendency to make equal allocations in the face of disliked individuals was relatively stronger. (2) Resource quantity had a significant hindering effect on the allocation behavior of sixth graders, when there were limited resources, children were more willing to make altruistic allocation. When there were abundant resources, children were more likely to make selfish allocations. An increase in resource quantities resulted in a lower proportion of children to make equal allocations. In other words, children were likely to make equal allocations when there were limited resources. (3) Considering that children's allocation behavior is influenced by various factors, apart from the influence of resource quantities and peer relationships on children's allocation behavior found in this study, it is also important to acknowledge that inequality aversion, different allocation contexts, and the level of theory of mind may affect the distribution of children to varying degrees. The implications of this research can be extended to how children navigate complex situations involving interpersonal relationships. Especially in real schools with imbalance educational resources, it is particularly important for pupils to maintain an equitable and impartial attitude toward resource allocation issues among peers, teachers, and families.

## Limitations and directions for future research

Several limitations of this study need to be noted. Firstly, according to the findings examined by Sebanc et al. (2003) gender differences appear to exist primarily in structures related to social competence itself, and sharing behavior, as part of pro-social behavior, is itself closely linked to children's moral cognition and moral behavior. Hallers-Haalboom et al. (2020) found that boys tended to share with friends and girls with acquaintances. Suggesting that gender differences also need to be attended to in similar research studies. Secondly, future experimental allocation procedures could be modified to involve children in both benefit-involved and non-benefit-involved scenarios, comparing children's sharing behaviors in different allocation modes. Third, previous studies have shown that the number of resources influences children's allocation behaviors. However, including our present study, most established studies still control for single-digit resource quantities. Therefore, it is recommended that future experiments increase the number of resources allocated in similar settings. Fourthly, human giving and taking of resources develops in interaction with significant others (Rheingold et al., 1976), and the importance of cooperation as a prerequisite for sharing cannot be ignored. Our experiments may have been limited by not allowing children to act solely as allocators. Both the dictator game and the ultimatum game could be used as allocation paradigms in the future, allowing children and recipients to interact and cooperate in resource allocation games under conditions of simultaneous “presence.” Lastly, the development of the theoretical level of the sixth-grade students should be included in the experiment, since 12-year-old children can already reconcile their own needs with the needs of others. Inferring the mental states of others and adapting one's own sharing behavior is very common among older children.

## Data availability statement

The original contributions presented in the study are included in the article/supplementary material, further inquiries can be directed to the corresponding author.

## Ethics statement

The studies involving humans were approved by the ShenJiaYing Primary School of Huangshi City, Hubei Province. The studies were conducted in accordance with the local legislation and institutional requirements. Written informed consent for participation in this study was provided by the participants' legal guardians/next of kin.

## Author contributions

RC: Investigation, Validation, Writing – original draft. HZ: Funding acquisition, Methodology, Resources, Writing – review & editing.
